# Camrelizumab in combination with doxorubicin, cisplatin, ifosfamide, and methotrexate in neoadjuvant treatment of resectable osteosarcoma: A prospective, single‐arm, exploratory phase II trial

**DOI:** 10.1002/cam4.70206

**Published:** 2024-09-26

**Authors:** Qinglian Tang, Xinke Zhang, Xiaojun Zhu, Huaiyuan Xu, Guohui Song, Jinchang Lu, Hao Wu, Chuangzhong Deng, Fei Ai, Yingchun Zhang, Jin Wang

**Affiliations:** ^1^ Department of Musculoskeletal Oncology Sun Yat‐Sen University Cancer Center Guangzhou China; ^2^ State Key Laboratory of Oncology in South China, Collaborative Innovation Center for Cancer Medicine Sun Yat‐Sen University Cancer Center Guangzhou China; ^3^ Department of pathology Sun Yat‐Sen University Cancer Center Guangzhou China; ^4^ Department of radiology Sun Yat‐Sen University Cancer Center Guangzhou China

**Keywords:** camrelizumab, neoadjuvant therapy, osteosarcoma, programmed cell death receptor 1

## Abstract

**Background:**

The poor overall survival of osteosarcoma (OS) underscores the need to explore new therapeutic avenues. Tumor necrosis rate (TNR) after neoadjuvant chemotherapy predicts prognosis.

**Aims:**

The study was to investigate safety and activity of neoadjuvant chemotherapy with camrelizumab (a humanized antibody against PD‐1) in patients with resectable OS.

**Materials & Methods:**

We conducted a prospective, single‐arm, exploratory phase II trial in OS patients. Eligible patients received camrelizumab combined with doxorubicin or liposomal doxorubicin, cisplatin, methotrexate, ifosfamide with mesna. Surgery was performed 12–14 days after neoadjuvant therapy and adjuvant therapy starting 2–3 weeks postoperatively. The primary endpoint was the rate of good tumor necrosis (TNR ≥90%) after neoadjuvant therapy, and the secondary outcomes were safety, 2‐year progression free survival and 2‐year overall survival.

**Results:**

Seventy‐five patients were recruited to the study. Subsequently, 64 patients completed neoadjuvant therapy and underwent surgery. Thirty‐one patients (48.4%) have a good TNR to neoadjuvant therapy. With a median follow‐up of 22.4 months (range 2.2–44.9 months), the estimated 2‐year PFS was 69.6% and the estimated 2‐year overall survival was 89.4%. Grade 3 or 4 treatment‐related adverse events were noticed in 62.7% of the patients. Frequent grade 3 or 4 adverse events were decreased platelet count (45.3%), decreased white blood cell count (36%). No immune‐related serious adverse events were observed.

**Discussion:**

Our study had limitations. First, it was limited by its non‐randomized design. Besides, stromal tumor‐infiltrating lymphocytes was comprehensively analyzed in this study.

**Conclusions:**

This study demonstrated that amrelizumab combined with adriamycin, cisplatin, methotrexate, and ifosfamide in the neoadjuvant treatment of resectable OS was safe and tolerable. This combined therapeutic strategy may not increase TNR, but the long‐term survival benefit remains to be followed up.

## 1 INTRODUCTION

Osteosarcoma (OS) is the most common primary bone sarcoma in children and young adults.^1^ The treatment for newly diagnosed OS typically is chemotherapy and surgical resection.^2^ The 5‐year survival rate is approximately 70% for localized disease, however, only 20% for metastatic cases.^3^, ^4^ There have been only modest changes in the standard of care since 2002, underscoring the need to explore new therapeutic avenues.

The tumor necrosis rate (TNR) after neoadjuvant chemotherapy predicts survival of OS patients.^5^ Studies reported that 30%–50% of patients had at least 90% necrosis after conventional neoadjuvant chemotherapy.^6^, ^7^, ^8^, ^9^ Patients with a good response (TNR ≥90%) survived longer than those responded poorly (TNR <90%).^7^ In addition, neoadjuvant therapy could render marginally inoperable tumors operable, facilitating limb‐salvage.

However, treatment options for increasing TNR are limited. Neoadjuvant combination therapies, including chemotherapy plus programmed cell death receptor 1 (PD‐1) antibody showed promising activity in several solid tumors.^10^ To date, antitumor activity of neoadjuvant chemotherapy plus PD‐1 antibody has not been investigated in resectable OS.

In fact, we previously found that neoadjuvant chemotherapy may stimulate anticancer immunity in OS.^11^ These findings indicated that it is worth investigating neoadjuvant chemotherapy and immunotherapy treatment in OS patients. Camrelizumab (AiRuiKa™), a humanized antibody against PD‐1, showed antitumor activity in various tumors.^12^, ^13^, ^14^

In this study, our aim was to evaluate the safety and antisarcoma activity of neoadjuvant chemotherapy with camrelizumab in OS patients.

## 2 METHODS

### 2.1 Study design and patients

This prospective, single‐arm, exploratory phase II trial was conducted at Sun Yat‐sen University Cancer Center. Informed consent was obtained in the study.

Eligible criteria included age 14 years or older, histopathologically confirmed high‐grade primary OS, at least one measurable disease by MRI according to Response evaluation criteria in solid tumors (RECIST) version 1.1, the ability to achieve wide resection after neoadjuvant chemotherapy, and ECOG Performance Status score of 0–1. Sufficient liver heart, bone marrow, kidney function, and endocrine function were required within 14 days before first camrelizumab administration.

Key exclusion criteria were active autoimmune disease, central nervous system metastases, and hypersensitivity to components of the camrelizumab formulation. Patients with prior treatment of PD‐1/PD‐L1 or CTLA‐4 antagonists were also excluded.

### 2.2 Procedures and outcomes

Eligible patients received neoadjuvant therapy with camrelizumab (200 mg or 3 mg/kg if the body weight ≤40 kg, Day 1, 22, 43), doxorubicin (37.5 mg/m^2^) or liposomal doxorubicin (25 mg/m^2^) on Day 1–2 and Day 43–44, cisplatin (100 mg/m^2^, Day 2 and 44), methotrexate (8–12 g/m^2^, Day 12), ifosfamide (3 g/m^2^, Day 22–25), and mesna (0.6 g/m^2^, Day 22–25).

Surgery was performed 12–14 days after neoadjuvant therapy completion, and adjuvant therapy was started 2–3 weeks postoperatively. Adjuvant therapy consisted of camrelizumab (200 mg or 3 mg/kg if the body weight ≤40 kg, Day 1, 22), methotrexate (10–12 g/m^2^, Day 2), doxorubicin (37.5 mg/m^2^) or liposomal doxorubicin (25 mg/m^2^) on Day 9–10, cisplatin 100 mg/m^2^ (Day 10) and ifosfamide 3 g/m^2^ (Day 22–25). This adjuvant regimen was repeated for three cycles.

Dose modifications for chemotherapy drugs due to adverse events were done according to the protocol. Dose modifications for camrelizumab were not allowed, but dose delays up to 12 weeks were considered for adverse events.

We adopted the National Cancer Institute Common Terminology Criteria for Adverse Events version 4.0 to grade adverse events during treatment and up to 30 days after treatment discontinuation.

The rate of good tumor necrosis (TNR ≥90%) after neoadjuvant therapy was the primary outcome, and the secondary outcomes were safety, 2‐year progression free survival (PFS), and 2‐year overall survival.

Surgical specimens were sent for pathological assessment of TNR. TNR was determined under the protocols previously reported.^15^, ^16^ Disease was assessed with MRI or CT scans at baseline, preoperatively, and then every 3 months postoperatively until disease progression. Radiologic response was evaluated by RECIST version 1.1.

Safety assessments, including laboratory monitoring, were carried out on the first day of each cycle of therapy.

### 2.3 Statistical analysis

According to the literatures and our previous clinical experiences, we hypothesized that combination of camrelizumab with doxorubicin, cisplatin, ifosfamide, and methotrexate would lead to a good TNR of 45% or higher. With confidence level of 0.95 and confidence interval width of 0.25, the sample size was 67 which was calculated by Clopper–Pearson methods. Seventy‐five patients with OS were finally included with estimated drop‐out rate of 10%.

Patients who received at least one dose of camrelizumab were included in the safety analysis. The categorical data were described as numbers and percentages. We used Kaplan–Meier method to estimate 2‐year PFS and 2‐year overall survival.

## 3 RESULTS

### 3.1 Patients characteristics

**FIGURE 1:**
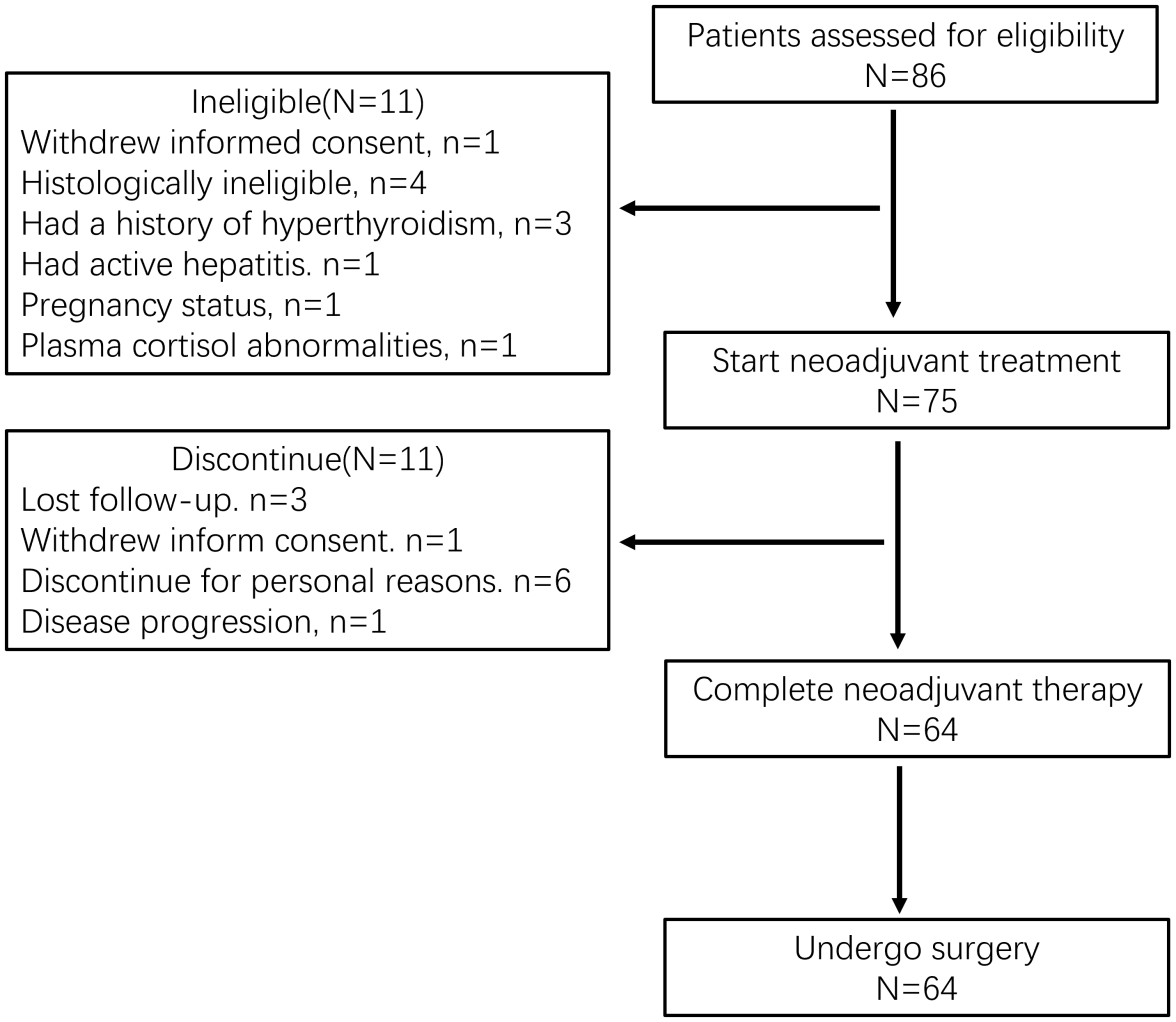
Flowchart of patient selection.

A total of 86 patients were screened and informed consent was signed in 75 patients from December 2019 to June 2022. Among them, 64 patients completed neoadjuvant therapy and underwent surgery (Figure 1).

Of the 64 patients, the median age was 17 years (range 14–50). Thirty‐four patients (53.1%) had primary tumors in the femur, 13 (20.3%) in the tibia, and 12 (18.8%) in the humerus. Fifty‐five (85.9%) patients were Enneking IIB, and 9 (14.1%) were Enneking III. Elevated baseline lactate dehydrogenase (LDH) and alkaline phosphatase (ALP) were detected in 30 (46.9%) and 19 (29.7%) of the patients, respectively. After surgery, 51 patients completed adjuvant treatment. The demographic and clinical characteristics are shown in Table 1.

**TABLE 1:** Patient characteristics (*n* = 64).

Gender, *n* (%)	
Male	49 (76.6%)
Female	15 (23.4%)
Age	
Median, years (range)	17 years (range 14–50)
Site, *n* (%)	
Femur	34 (53.1%)
Tibia	13 (20.3%)
Humerus	12 (18.8%)
Pelvis	3 (4.7%)
Fibula	1 (1.5%)
Radius	1 (1.5%)
Enneking stage, *n* (%)	
IIB	55 (85.9%)
III	9 (14.1%)

### 3.2 Treatment and response

**FIGURE 2:**
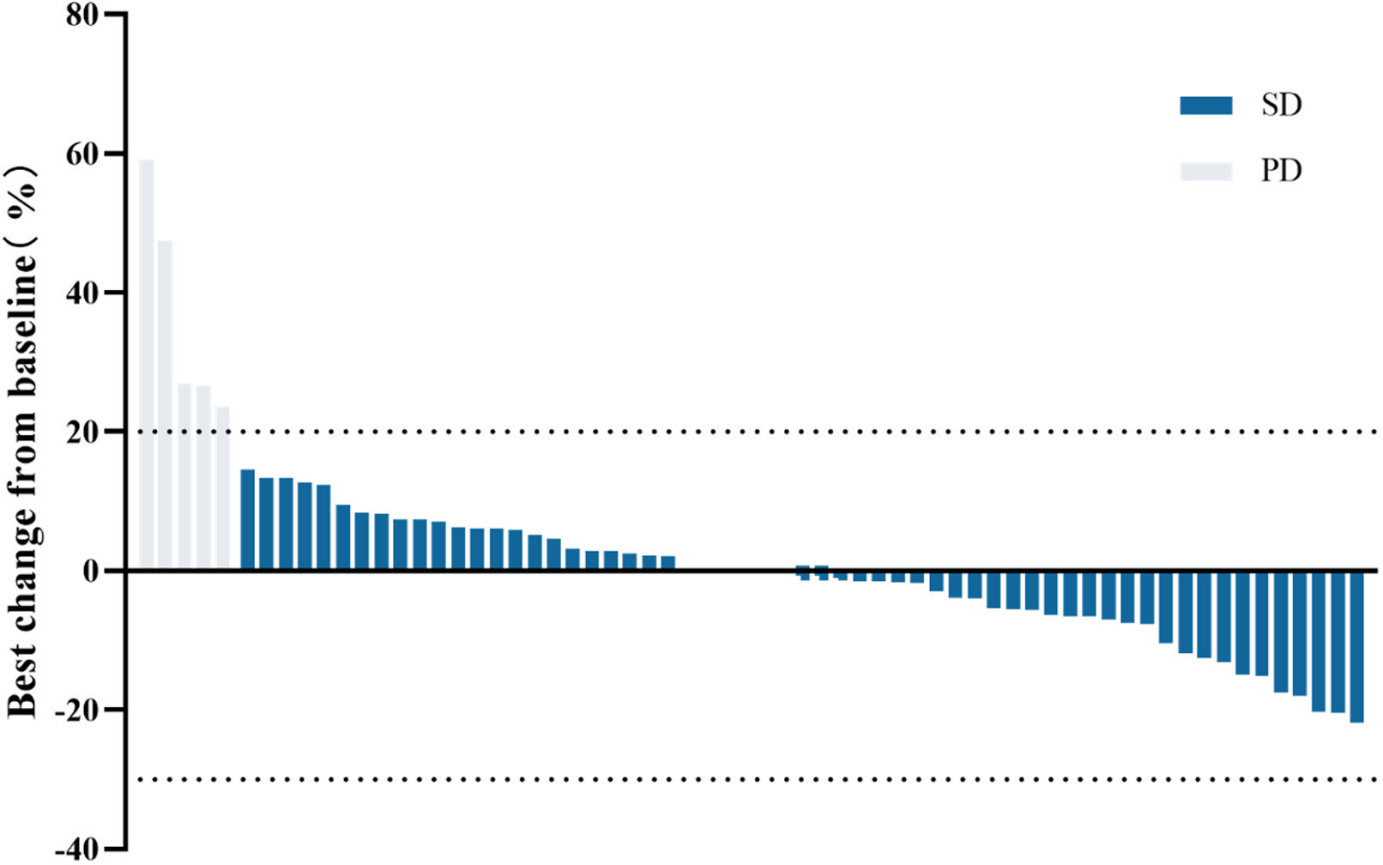
Best percentage change from baseline.

After completing neoadjuvant therapy, five patients were classified as having progressive disease (PD) and the remaining 59 patients were having stable disease (SD) after assessment with RECIST version 1.1. The disease control rate (CR/PR/SD) was 92.2%. Best overall response is shown in Figure 2.

**FIGURE 3:**
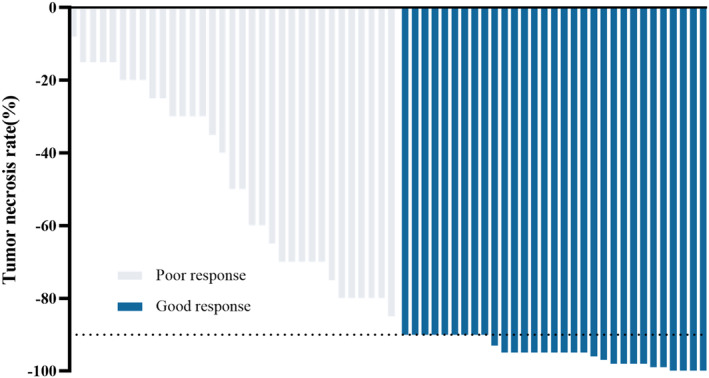
Tumor necrosis rate (TNR) after neoadjuvant therapy.

All the 64 patients received surgical resection and were evaluable for the primary outcome, among which one patient received amputation, and the remaining 63 patients had limb‐salvage surgery. Thirty‐one (48.4%) patients had a good response to neoadjuvant therapy, which was indicted by a TNR ≥90% (Figure 3). No significant difference in TNR was found between patients younger than 18 years old and that aged 18 and older. Also, no correlation between baseline ALP or LDH with TNR was found.

### 3.3 Survival outcome

**FIGURE 4:**
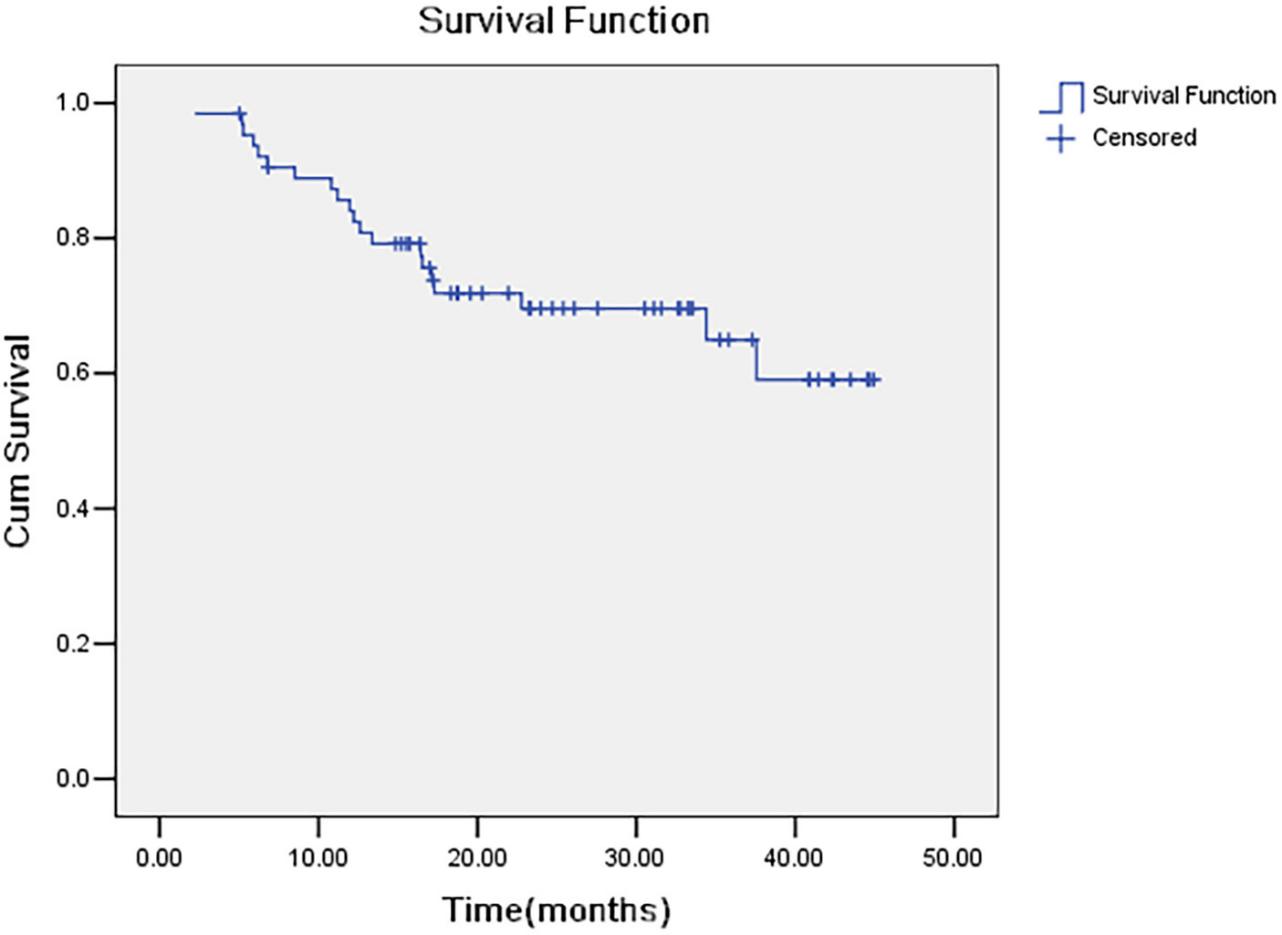
Progression free survival (PFS).

With a median follow‐up of 22.4 months (range 2.2–44.9 months), 20 (31.3%) of 64 evaluable patients had a progression event (18 progressed and 2 died). The estimated 2‐year PFS was 69.6% (95% CI 57.6%–81.6%) (Figure 4).

**FIGURE 5:**
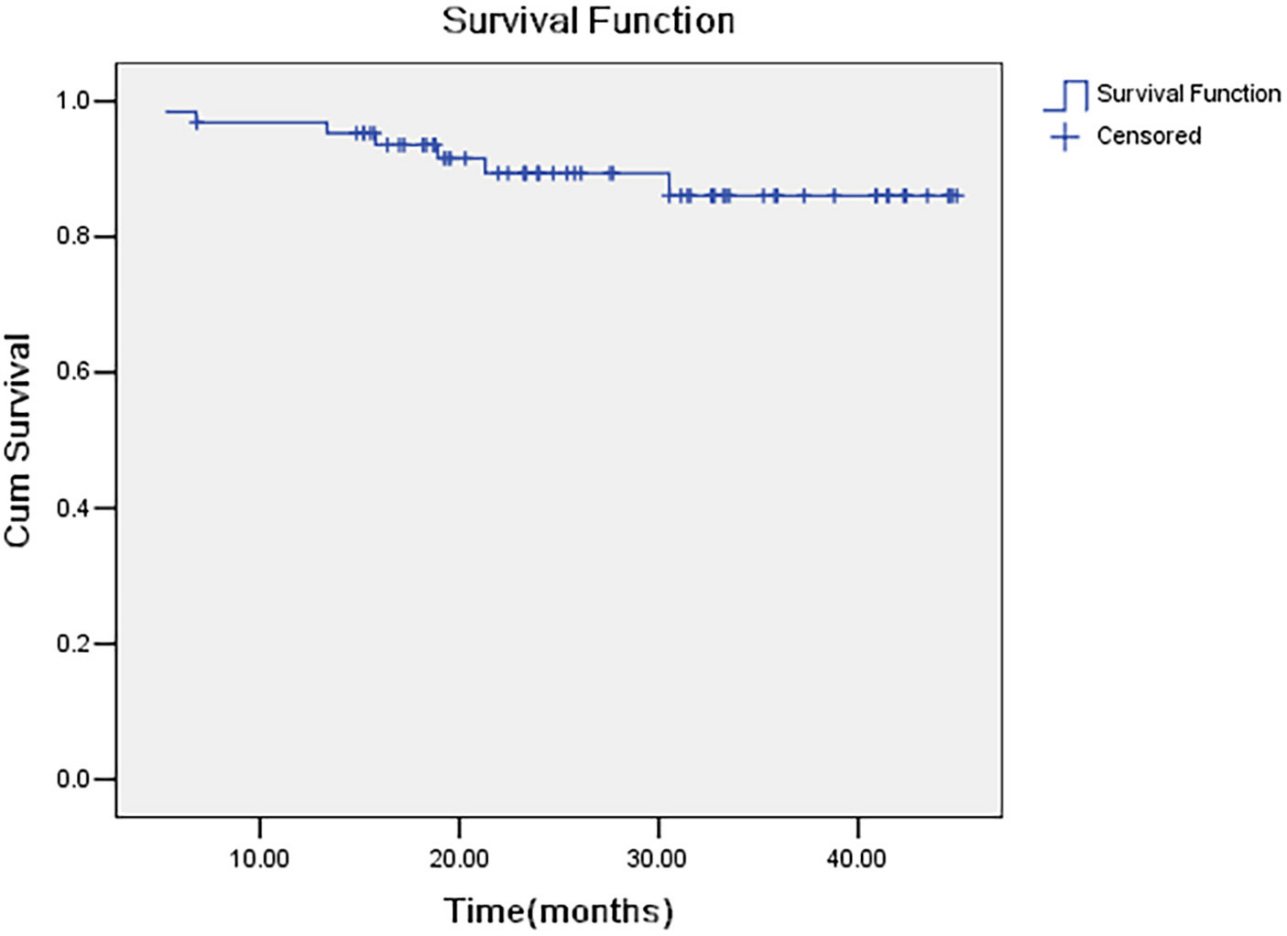
Overall survival (OS).

At the time of last visit, 57 (89.1%) of patients were alive. The estimated 2‐year overall survival was 89.4% (95% CI 81.2%–97.6%) (Figure 5).

### 3.4 Adverse events

Patients receiving at least one dose of camrelizumab and chemotherapy were included for adverse event analysis. All adverse events first occurred during neoadjuvant chemotherapy. The grade 3 or 4 treatment‐related adverse events were noticed in 62.7% of the patients. The most frequent grade 3 or 4 adverse events were decreased platelet count (34 [45.3%]), decreased white blood cell count (27 [36%]), oral mucositis (10 [13.3%]), and increased alanine aminotransferase (9 [12%]). No immune‐related serious adverse events were observed One patient discontinued camrelizumab treatment because of drug allergy. One patient died of severe myelosuppression after treatment. (Table 2).

**TABLE 2:** Adverse events related to treatment.

Adverse events (AEs)*	Grade1‐4, *n* (%)	Grade 3–4, *n* (%)
Hematology		
White blood cell counts decreased	62 (82.7)	27 (36.0)
Platelet count decreased	59 (78.7)	34 (45.3)
Neutrophil count decreased	57 (76.0)	23 (30.7)
Anemia	9 (12.0)	4 (5.3)
Non‐hematology		
Vomiting	55 (73.3)	0 (0.0)
Alanine aminotransferase increased	56 (74.7)	9 (12.0)
Aspartate aminotransferase increased	51 (68.0)	8 (10.7)
Nausea	37 (49.3)	0 (0.0)
Mucositis oral	26 (34.7)	10 (13.3)
Pyrexia	22 (29.3)	2 (2.7)
Reactive capillary endothelial proliferation	19 (25.3)	0 (0.0)
Vertigo	12 (16.0)	0 (0.0)

## 4 DISCUSSION

To our knowledge, this is the first clinical trial to explore the safety and activity of neoadjuvant chemotherapy combined with immunotherapy in patients with OS. Our study showed that camrelizumab combined with adriamycin, cisplatin, methotrexate, and ifosfamide in the perioperative treatment of OS patients was safe and tolerable. Addition of camrelizumab to standard neoadjuvant chemotherapy did not increase toxicity, as compared with previous reports.^3^, ^7^, ^17^, ^18^

No CR/PR was recorded by RECIST criteria in our study. This result was consistent with previous reports.^19^, ^20^ Guenther et al found no patients achieved CR/PR response in the primary OS.^19^ Also, no association between RECIST response and pathologic necrosis was found in our study and other studies.^19^, ^20^ Current data revealed that RECIST criteria may be not sensitive enough to determine the treatment response of OS in the neoadjuvant setting. However, our study and other studies confirmed that patients with PD had worse prognosis than patients with SD or PR (Figure S1).

We examined the pathologic response in surgical specimen for neoadjuvant PD‐1 blockage, using TNR as an indicator. TNR after neoadjuvant therapy is a well‐recognized clinical biomarker for prognosis in OS. In the present study, patients with higher TNR have better survival, although this did not reach statistical significance due to the relatively small sample size and limited time of follow‐up (Figure S2). According to the literatures, good response rate was around 40%–65% after neoadjuvant therapy.^3^, ^8^, ^17^, ^21^ In our study, the good response rate was 48.4%, indicating that this combination regimen may not increase TNR compared with conventional neoadjuvant chemotherapy. Our results suggested that PD‐1 antibody may have limited added antitumor activity in the neoadjuvant chemotherapy setting. In fact, several previous studies found that PD‐1 antibody alone had low activity in advanced OS.^22^, ^23^, ^24^, ^25^ The mechanisms of immunotherapy resistance remain to be investigated. We detected expression of PD‐L1 in the surgical specimens after neoadjuvant chemotherapy and found no staining of PD‐L1 in the tumor cells (data not shown).Complex tumor‐immune microenvironment and low PD‐L1 expression in OS may be one possible reason for unsatisfactory treatment response of PD‐1 blockade.^26^, ^27^ Besides, unique machinery of antigen processing and presentation and the lack of neoantigens to be attacked may also impede the effect of immunotherapy. Approaches to enhance systemic activation of tumor‐specific T cells and identification of neoantigen are needed for further investigation.

Apart from TNR, survival might be a better clinically relevant end point in OS trials. The estimated 2‐year PFS and OS in the present study was 69.6% and 89.4%, respectively, which were similar to previous reports.^4^ However, the effect of neoadjuvant immunotherapy combined with chemotherapy on long‐term survival remains to be followed up.

There is gender bias in the present study, as compared with previously reports.^16^, ^28^ The possible reasons were that only patients older than 14 years were included and the sample size of the study was relatively small.

Our study had limitations. First, it was limited by its non‐randomized design. Besides, stromal tumor‐infiltrating lymphocytes was comprehensively analyzed in this study. In the present study, intratumoral CD8^+^ TILs density evaluated by immunohistochemistry was 210 ± 220 (mean ± SD), which is much lower than PD‐1‐sensitive melanoma.^29^ However, this is the first study to assess the impact of neoadjuvant chemotherapy combined with PD‐1 blockage in OS using an in‐vivo parameter.

In conclusion, our study indicate that this therapeutic strategy is well‐tolerated in OS patients, with only modestly enhanced activity in TNR. However, the long‐term survival benefit remains to be followed up.

## AUTHOR CONTRIBUTIONS

**Qinglian Tang:** Formal analysis (equal); writing – original draft (equal). **Xinke Zhang:** Formal analysis (equal). **Xiaojun Zhu:** Investigation (equal). **Huaiyuan Xu:** Investigation (supporting). **Guohui Song:** Writing – review and editing (supporting). **Jinchang Lu:** Investigation (supporting). **Hao Wu:** Investigation (supporting). **Chuangzhong Deng:** Investigation (supporting). **Fei Ai:** Formal analysis (lead). **Yingchun Zhang:** Conceptualization (equal). **Jin Wang:** Conceptualization (equal); supervision (equal); writing – review and editing (equal).

## CONFLICT OF INTEREST STATEMENT

No conflicts of interest needed to disclose.

## ETHICS STATEMENT

The study was approved by the institutional review board of Sun Yat‐sen University Cancer Center and the registered number was NCT04294511.

## DISCLOSURE

The abstract was previously posted on ASCO 2023 (DOI https://doi.org/10.1200/JCO.2023.41.16_suppl.115).

## Supporting information


Figure S1.
Figure S2.

## Data Availability

The data in this study can be requested upon reasonable use.
