# Supplementary material for: Camrelizumab in combination with doxorubicin, cisplatin, ifosfamide, and methotrexate in neoadjuvant treatment of resectable osteosarcoma: A prospective, single‐arm, exploratory phase II trial
Source: Cancer Med. 2024 Sep 26;13(18):e70206. doi: 10.1002/cam4.70206 (PMC11424980; doi:10.1002/cam4.70206)
Supplement: Supplementary file 1 — Figure S1. Figure S2. [file CAM4-13-e70206-s001.docx]

**Supplementary Figure 1. Survival analysis between patients with progressive disease (PD) and stable disease (SD) (P=0.007).**

**Supplementary Figure 2. Survival analysis between patients with good TNR and poor TNR (P=0.072).**
